# Caregivers’ willingness and factors influencing uptake of the malaria vaccine among under-five children at Masaka Regional Referral Hospital, Uganda

**DOI:** 10.1371/journal.pgph.0005764

**Published:** 2026-10-06

**Authors:** Annet Mary Namusisi, Richard Migisha, Dominic Kizza, Benon Kwesiga, Lilian Bulage, Valeria Tusuubira, Lucy Maria Nakayiza, Sophia Tageya, Fred Nsubuga, Rita Atugonza, Michael Baganizi, Alex Riolexus Ario

**Affiliations:** 1 Uganda Public Health Fellowship Program, Uganda National Institute of Public Health, Kampala, Uganda; 2 Department of Data, Makerere University John Hopkins University, Kampala, Uganda; 3 Maternal and Child health Department, Masaka Regional Referral Hospital, Masaka, Uganda; 4 Uganda National Expanded Program on Immunization, Ministry of Health, Kampala, Uganda; Universiti Malaya, MALAYSIA

## Abstract

Uganda ranks third with the highest malaria cases globally, highlighting limitations in existing prevention efforts. Malaria vaccination is a key promising strategy to reduce both morbidity and severity among children under 5 (U5) years. In April 2025, Uganda introduced a four-dose R21 malaria vaccine for U5s. We assessed willingness to vaccinate with the malaria vaccine and associated factors among caregivers of U5s to support vaccine rollout in the targeted population. In March 2025, we conducted a cross-sectional study at the Maternal and Child Health unit of Masaka Regional Referral Hospital among 292 caretakers. We generated caretaker attendance lists daily to serve as sampling frames and developed a sampling interval. We used systematic sampling to obtain the total sample size. We administered a questionnaire assessing caregivers’ willingness to vaccinate, and identified associated factors using modified Poisson regression. We interviewed 292 caretakers, 250(86%) were female and 249(85%) indicated willingness to vaccinate their children. Prior experience with adverse events following immunization (AEFIs) (adjusted prevalence ratio [aPR]=0.2,95%Confidence Interval [CI]0.05-0.5), having other relatives than biological parents as main decision makers (aPR 0.2, 95%CI0.04-0.9) and having tertiary education (aPR 0.14, 95%CI0.04-0.6) were associated with reduced willingness to vaccinate, while receipt of malaria Intermittent Preventive Treatment in pregnancy (IPTp) (aPR 6.2, 95%CI1.6-23) and trust in vaccine safety (aPR 13, 95%CI4.0-43) were associated with increased willingness to vaccinate. Majority of the caretakers were not aware of the malaria vaccine butwere willing to vaccinate their children when oriented. AEFI history, household decision-making roles, and education level were associated with reduced willingness, while having received malaria IPTp and trust in vaccine safety were associated with increased willingness to vaccinate. We recommended providing malaria vaccine information through antenatal care, preparing health workers to address the AEFI history and vaccine-safety concerns and prioritizing safety-focused messaging over general awareness messaging.

## Background

Malaria remains one of the most devastating infectious diseases globally. In 2023, the World Health Organization (WHO) estimated 263 million malaria cases worldwide, with 94% of these occurring in WHO African region and 76% of these being children under 5 years of age [1]. Five countries - Nigeria, the Democratic Republic of Congo, Uganda, Ethiopia, and Mozambique accounted for 52% of this burden [1].

In Uganda, malaria is the leading cause of morbidity and mortality, with an estimated 95% of the population at risk [2]. According to the World Malaria Report 2024, Uganda ranked third globally among countries with the highest malaria burden in 2023, accounting for approximately 5% of all global malaria cases [1]. Between 2010 and 2023, the country recorded an increase of 1.3 million malaria cases, with Plasmodium falciparum species responsible for 97% of infections [1].

In response to the escalating malaria burden, the WHO approved two malaria vaccines for use in endemic countries. The first, RTS, S/AS01 (RTS, S), was recommended in 2021 to prevent severe disease and death in young children [3,4]. This was followed in 2023, by a second vaccine, R21/ Matrix-M(R21), recommended in a four-dose schedule for children in areas of moderate to high malaria transmission [5]. Integrating malaria vaccination into routine malaria control efforts was hoped to reduce inequities and overall disease burden among children, with projections estimating that malaria vaccination could avert up to half a million deaths by 2035 [6]. In line with this global effort, Uganda launched its malaria vaccine introduction campaign in April 2025, integrating R21/Matrix-M into the routine immunization program to substantially reduce malaria prevalence, particularly in the country’s moderate to high-burden regions (Uganda malaria vaccine introduction plan 2024─2026).

The success of malaria vaccination efforts relies on community willingness to vaccinate children [7]. Vaccine hesitancy, defined by the WHO as reluctance or refusal to vaccinate despite the availability of vaccines, has been rising in low- and middle-income countries, driven by misinformation, negative experiences at health facilities, and social marginalization [8,9]. Improving vaccine acceptance and childhood immunization coverage in Uganda therefore requires public health programs designed to address the specific barriers faced by diverse populations and communities.

Masaka City and District lie within a moderate malaria transmission zone according to the 2025 Uganda malaria indicator survey [10]. Malaria remains the leading cause of outpatient attendance at Masaka Regional Referral Hospital (MRRH), and although U5 mortality at the hospital declined from 55 to 36 deaths per 1,000 live births between 2020 and 2024, this rate remains above the Sustainable Development Goal (SDG) 3.2 target of fewer than 25 deaths per 1,000 live births by 2030. This underscores the continued vulnerability of U5 children in the region (DHIS2) [11,12]. As a key referral centre for severe malaria cases, MRRH offered an ideal setting to assess caregiver readiness for malaria vaccination ahead of the national rollout.

This study therefore assessed caregivers’ awareness of and willingness to vaccinate their children with the malaria vaccine, and the factors influencing this willingness among caregivers of children under 5 years attending MRRH before the national rollout. The findings supported evidence-based insights to guide malaria vaccine introduction strategies and maximize uptake in comparable transmission settings across Uganda.

### Conceptual framework for factors influencing caregivers’ willingness and uptake of the malaria vaccine among children under 5 years

Caregivers’ willingness and uptake of the malaria vaccine is shaped by three interactive domains, adopted from the Health Belief model and Behavioural Model of Health services.

Predisposing factors represent the cognitive and attitudinal conditions that shape a caregiver’s initial orientation toward vaccination, including awareness of the malaria vaccine, knowledge about malaria, prior vaccination history, trust in the health system and cultural or religious beliefs that may either facilitate or impede acceptance [13,14].

Enabling factors represent the structural and social conditions that translate willingness into actual uptake, including access to health facilities, vaccine availability, sensitization, and health worker recommendations. These factors determine whether or not a caregiver will vaccinate their child despite their willingness [14,15].

At the individual level, a caregiver’s malaria awareness, perceived severity of malaria, perceived benefit of vaccination, and degree of vaccine hesitancy directly influence their decision to vaccinate [16]. At the interpersonal level, healthcare worker guidance, community norms, cultural and religious beliefs, and exposure to misinformation either reinforce or undermine acceptance [17]. At the health system level, accessibility of vaccination services, health system trust, and vaccine availability determine whether willingness is converted into actual uptake [15].

There may be confounders such as socio-demographic factors- caregiver age, parity, socio-economic status, and place of residence- that affect willingness and vaccine uptake. Older caregivers may have greater experience with childhood illnesses and vaccinations, while younger caregivers may be more exposed to misinformation circulating on social media platforms [18]. Socioeconomic status affects a caregiver’s ability to meet indirect costs of accessing healthcare, and rural caregivers face greater geographical barriers and more limited access to accurate health information than their urban counterparts [19]. In addition, controlling confounders would provide adequate grounds to establish the actual factors that affect uptake of the malaria vaccine (Fig 1).

**Fig 1:**
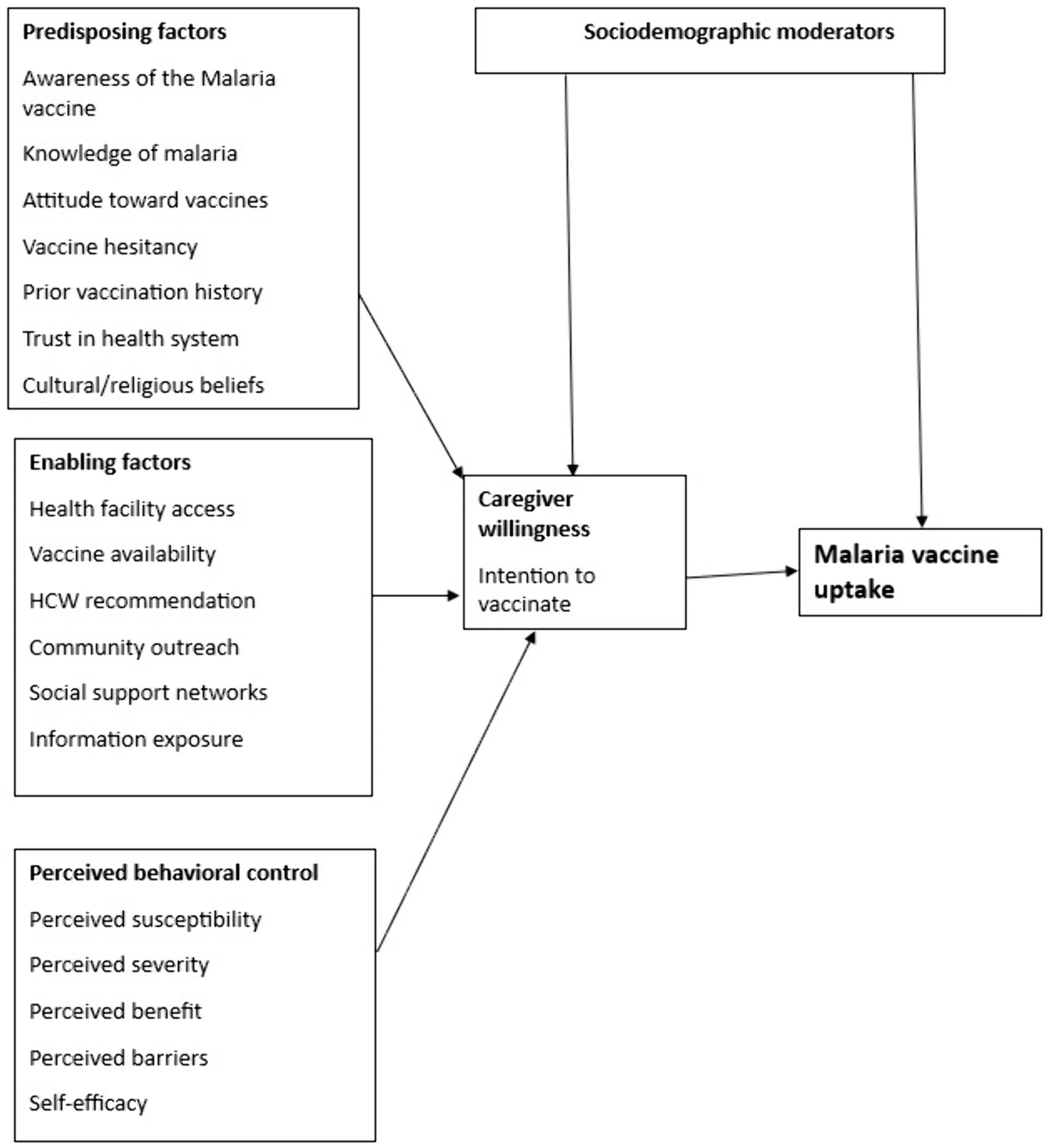
Conceptual framework for factors influencing caregivers’ willingness to vaccinate U5s with the malaria vaccine (adopted from the Health Belief model and Behavioural Model of Health services).

## Methods and materials

### Ethics statement

The study was in response to a public health emergency and was therefore determined to be non-research. The Ministry of Health (MoH) gave permission to carry out this study to obtain the community's perception of the ongoing new vaccine introduction in the country. In agreement with the International Guidelines for Ethical review of Epidemiological studies by the Council for International Organizations of Medical Sciences (1991) and the Office of the Associate Director for Science, US CDC/Uganda, it was determined that this activity was not human subject research and its primary intent was public health practice specifically endemic disease control activity. This investigation was reviewed by the US CDC and was conducted consistent with applicable US federal laws and CDC policy. § §See, e.g., 45 C.F.R. part 46, 21 C.F.R. part 56; 42 U.S.C. §241(s); 5 U.S.C. §552a; 44 U.S.C. §3501 et seq. All experimental protocols were approved by the US CDC human subjects review board (The National Institute for Occupational Safety and Health Institutional Review Board), and Uganda MoH and were performed in accordance with the Declaration of Helsinki. We did not obtain ethical approval from the Ugandan Institutions Ethics Committee but obtained permission to conduct the Epidemiological study from the Masaka Regional Referral Hospital administration. We further obtained written informed consent from each participant before conducting any interviews. Only participants 18 years and above, and no minors, were considered for the study. All participant records were anonymized to protect from participant identification and maintain confidentiality.

### Study setting and design

We conducted a cross-sectional study at the Maternal and Child Health Unit (MCH) of Masaka Regional Referral Hospital (MRRH) in south-central Uganda. The MCH clinic offers a range of services including: antenatal care, immunization, prevention of mother-to-child HIV transmission, family planning, and delivery care. As a public facility supported by the Uganda Ministry of Health, MRRH provides healthcare free of charge and serves as a referral center for nine districts. In 2024, the Masaka Region had a population of U5 children of 209,150 with vaccination coverage of 67% (DHIS2) and the hospital conducts an average of 250 childhood vaccinations per week.

### Study population, sampling, and sample size considerations

The study population comprised caregivers of children under five years attending the MCH clinic during the study period. We defined a caregiver as a legal guardian, biological parent or other adult responsible for the well-being of the child including meeting the physical, emotional, social, and cognitive needs from birth [20].

To be eligible for participation, caregivers had to meet the following inclusion criteria: being a caregiver of a child aged under 5 years, being 18 years of age or older, and having received or currently receiving vaccination services for their child at the study facility during the study period. Caregivers who were mentally incapacitated and therefore unable to provide informed consent or respond reliably to study questions asked were excluded from the study.

The required sample size was estimated based on the Kish-Leslie formula for single proportions, assuming a 95% level of confidence level, a proportion of respondents aware of the malaria vaccine of 18% [21], and 5% precision. $N=\frac{P(\text{1}-P)Z^{\text{2}}}{D^{\text{2}}}$, where N is the required sample size, p is the proportion of caregivers aware of the malaria vaccine, and d is the margin of error. Z is the standard normal deviate, 1.96 (the Z-score corresponding to a 95% confidence interval). Accounting for an anticipated 10%non-response rate, the final sample size was 226+ (10% of 226) = 292 respondents.


$$\begin{array}{c} \text{N}=\;\frac{\text{0}.\text{18}(\text{1}-\text{0}.\text{18})\;{\text{1}.\text{96}}^{\text{2}}}{{\text{0}.\text{05}}^{\text{2}}}=\text{226 }+(\text{10}\%\;\times \text{226})=\text{29}\text{2}\text{ respondents} \end{array}$$

Participants were selected using systematic random sampling. Each day, an attendance list of caregivers whose children were present for immunization was compiled and used as the sampling frame. The daily enrollment target was calculated by dividing the total sample size by the 14-day study period (292/14 = 21). We developed a sampling interval by dividing the number of attendees in the sampling frame by the daily enrollment target of 21, and the nth caregiver on the list was selected accordingly. This process was repeated daily until the overall sample size of 292 was reached.

### Study variables and data collection

Data were collected by trained healthcare workers (nurses) using a semi-structured, interviewer-administered questionnaire, part of which was adapted from the Behavioral and Social Drivers for vaccination (BeSD) tool [22]. The dependent variable, *caregivers’ willingness to vaccinate their child with the malaria vaccine*, was defined as the caregiver’s intention or readiness to have their child vaccinated and was assessed with the question *“Are you willing to vaccinate your child with the malaria vaccine?”* Awareness of the malaria vaccine was assessed with the question: *“Have you ever heard about the malaria vaccine?”*

Independent variables included caregiver and child sociodemographic and child-related characteristics: age, gender, education level, marital status, primary caregiver, relationship to the child, place of residence, gender of the child, number of children under five years, history of fever or admission, whether the child had ever suffered from malaria, knowledge about malaria, difficulty accessing vaccination services, presence of comorbidities, awareness about the malaria vaccine, perceptions of vaccine safety and effectiveness, presence of religious or cultural beliefs against vaccination, and experience of adverse events following immunization (AEFIs). Notably, AEFIs were caregiver-reported and not clinically confirmed, given the lack of adequate clinical documentation and included symptoms such as fever, pain, and swelling at the injection site, and irritability. Caregivers who reported AEFIs had not brought them to the attention of the health facility as the reactions were minor and resolved within a day of vaccination.

Data were collected during 3^rd^ ─17^th^ March 2025 using the electronic Kobo Collect tool. To ensure data quality, the questionnaire was pretested at Kalisizo General Hospital, the main referral hospital serving over 29 health facilities in Kyotera District. The hospital’s maternal and child health (MCH) clinic receives 60–90 clients daily for services such as immunization, antenatal care, and family planning. Kalisizo was selected for pretesting because its population shares similar sociodemographic and healthcare characteristics with the study setting, enabling assessment of question clarity and relevance. Before data collection, all data collectors underwent training on study tools and interviewing techniques to ensure accuracy, consistency, and adherence to ethical standards during data collection. After data cleaning, 292 children were included in the final analysis. Missing data were present in a small number of variables, such as religious and cultural beliefs, where participants declined to respond. However, they were assessed to be low < 2%, missing completely at random, and were not related to willingness to vaccinate. Complete case analysis (list-wise deletion) was done, and only complete data on relevant variables were included in the analysis. This provided unbiased parameter estimates.

### Data management and analysis

Data were analyzed using *Stata version 15 (StataCorp, College Station, TX, USA).* We used descriptive statistics to summarize participants’ characteristics and key study variables, presented as means with standard deviations (for continuous variables) and frequencies with corresponding percentages (for categorical variables). The primary outcome variable-caregivers’ willingness to vaccinate their child against malaria—was binary (yes/no). Given the high prevalence (85%) of willingness to vaccinate, modified Poisson regression with robust standard errors was used to estimate prevalence ratios (PRs) and their 95% confidence intervals (CIs), instead of odds ratios, to avoid overestimation of the association. Robust standard errors accounted for potential heterogeneity of variance across comparison groups. At the bivariate level, associations between each independent variable and the outcome were assessed using crude prevalence ratios (cPR). Before multivariate analysis, multicollinearity among independent variables was assessed using the Variance Inflation Factor (VIF). Variables with a VIF > 5 were considered collinear and were eliminated or combined with related variables to improve model stability. At model building, independent variables with *p*-values <0.20 were included for backward elimination in the multivariable model while controlling for potential confounding factors such as age and sex. Their adjusted prevalence ratios (aPR) were obtained. We used the Hosmer-Lemeshow test to determine the model’s goodness of fit. Statistical significance was determined at *p* < 0.05.

### Data quality

We used experienced research assistants (RAs)- qualified assistant nursing officers from Kalisizo General Hospital, who had prior experience in health facility-level data collection. Using RA s external to MRRH, rather than MRRH’s own health workers, was intended to minimize response bias that would arise from the respondents when interviewed by staff involved in their child’s care. The research assistants were trained on the protocol and all study tools for one day, participated in piloting the data collection tools, and were oriented in good clinical practice prior to data collection.

## Results

### Socio-demographic characteristics of caregivers of children under 5 years at Masaka Regional Referral Hospital, Uganda

Most children were under one year of age, 179 (61%). The majority of the caregivers were biological mothers, 261 (89%), and primary caregivers, 282 (97%), with the majority residing in urban areas, 180 (61%). Nearly half had attained a secondary level of education, 131 (45%). Most caregivers were very knowledgeable about malaria transmission, 175 (60%), and nearly all reported that their children slept under a mosquito net, 280 (96%) (Table 1).

**Table 1:** Socio-demographic characteristics of caregivers of children under 5 years at Masaka Regional Referral Hospital, Uganda, March 2025 (N = 292).

Variable	n (%)
**Child age (years)**	
<1	179 (61.3)
1-2	67 (22.9)
3-4	27 (9.3)
>4	19 (6.5)
**Sex of caregiver**	
Male	42 (14.4)
Female	250 (85.6)
**Education level**	
None	6 (2.0)
Primary	104 (35.6)
Secondary	131 (44.9)
Tertiary/University/College	51 (17.5)
**Religion**	
Catholic	127 (44)
Pentecostal	48 (16)
Anglican	47 (16)
Muslim	70 (24)
**Residence**	
Urban	180 (61)
Rural	112 (38)
**Relationship to child**	
Other relative	15 (5.1)
Mother	261 (89.4)
Father	16 (5.5)
**Main decision maker**	
Mother	168 (58)
Father	109 (37)
Other family member	15 (5)
**Primary caregiver**	
No	10 (3)
Yes	282 (97)
**Number of children <5**	
1	185 (63.4)
2	78 (26.7)
3	22 (7.5)
4	7 (2.4)
**Distance from Health center (Km)**	
0-5	161 (55.1)
6-10	25 (8.6)
11-15	52 (17.8)
>15	54 (18.5)
**Knowledge of malaria spread**	
No knowledge	28 (9.6)
Knowledgeable	89 (30.5)
Very knowledgeable	175 (59.9)
**Difficulty accessing childhood vaccinations**	
No	229 (78)
Yes	63 (22)
**Child sleeps under a mosquito net.**	
No	13 (4.5)
Yes	279 (95.5)

### Awareness and willingness to vaccinate with the malaria vaccine among caregivers of children under 5 at Masaka Regional Referral Hospital, Uganda, March 2025

Of the 292 caregivers interviewed, more than half were not aware of the malaria vaccine, 169 (58%), though the majority, 249 (85%) were willing to have their children vaccinated when oriented about the vaccine. Most caregivers, 229(78%), had no difficulty accessing their routine vaccinations and 261(89%) indicated they would purchase the malaria vaccine at $3–4(UGX10,500–14,000) per dose if it were available for sale. Overall, 236(81%) considered the malaria vaccine safe and effective for use among children U5 (Table 2).

**Table 2:** Awareness and willingness to vaccinate with the malaria vaccine among caregivers of children under 5 years at Masaka Regional Referral Hospital, Uganda, March 2025 (N = 292).

Variables (N=292)	n (%)
**Caregivers ever heard about the malaria vaccine**	
Yes	123 (42)
No	169 (58)
**Difficulty accessing child vaccination**	
Yes	63 (22)
No	229 (78)
**Caregivers could buy the malaria vaccine if available in the country**	
Yes	261 (89)
No	31 (11)
**Caregivers think malaria vaccines are effective in preventing malaria**	
Yes	236 (81)
No	56 (19)
**Caregivers think malaria vaccines are safe for their children**	
Yes	236 (81)
No	56 (19)
**Caregivers are willing to vaccinate their children with the malaria vaccine**	
Yes	249 (85)
No	43 (15)

### Factors associated with willingness to vaccinate with the malaria vaccine among caregivers of children under 5 years at Masaka Regional Referral Hospital, Uganda, March 2025

Of the 292 caregivers interviewed, 43 (15%) were not willing to vaccinate their child against malaria. At bivariate analysis, being the main decision maker, education level, relationship to the child, knowledge of how malaria spreads, receiving malaria Intermittent Preventive Treatment in pregnancy (IPTp) among the 250 female participants, perceived vaccine safety and effectiveness, and discouragement by religious or cultural beliefs were significantly associated with willingness to vaccinate (Table 3).

**Table 3:** Factors associated with willingness to vaccinate with the malaria vaccine among caregivers of U5 children at Masaka Regional Referral Hospital, Uganda, March 2025 (N = 292) [Bivariate analysis].

	Not willing to vaccinate	Willing to vaccinate			
**Characteristic**	**n (%)**	**n (%)**	**P-value**	**cPR**	**95% CI**
**Caregiver age (years)**					
15-24	19 (44)	93 (37)		Ref	
25-48	19 (44)	126 (51)	0.4	1.05	(0.94-1.16)
>48	5 (12)	30 (12)	0.7	1.03	(0.88-1.2)
**Level of Education**					
Primary	12 (28)	98 (39)		Ref	
Secondary	19 (44)	112 (45)	0.4	0.96	(0.87-1.06)
Tertiary/University	12 (28)	39 (16)	0.071	0.86	(0.73-1.01)
**Consider malaria vaccine effective**					
No	21 (49)	35 (14)		Ref	
Yes	22 (51)	214 (86)	<0.001	5.8	(2.9-12) **
**Child ever experienced Adverse Events Following Immunization (AEFI)**					
No	31 (72)	205 (82)		Ref	
Yes	12 (28)	44 (18)	0.1	0.6	(0.3-1.2)
**Discouragement by cultural/ religious beliefs about vaccination**					
Yes	24 (56)	177 (71)	0.05	0.5	(0.3-1.0)
No	19 (44)	72 (29)		Ref	
**Main decision maker**					
Myself/mother	26 (60)	142 (57)		Ref	
Spouse/partner	11 (26)	98 (39)	0.2	1.6	(0.8-3.5)
Other family member	6 (14)	9 (4)	0.02	0.3	(0.09-0.8) **
**Distance to the Health Centre (Km)**					
0-5	25 (58)	136 (55)		Ref	
6-10	5 (12)	20 (8)	0.6	0.7	(0.3-2.1)
10-15	4 (9)	48 (19)	0.2	2.2	(0.7-6.7)
>15	9 (21)	45 (18)	0.8	0.9	(0.4-2.1)
**Knowledge about malaria spread**					
No knowledge	1 (2)	27 (11)		Ref	
Knowledgeable	23 (54)	66 (26)	0.03	0.1	(0.01-0.8) **
Very knowledgeable	19 (44)	156 (63)	0.3	0.3	(0.04-2.4)
**Received malaria Intermittent Preventive Treatment in Pregnancy (IPTp)**					
No	8 (22)	11 (5.6)		Ref	
Yes	29 (78)	202 (95)	0.04	1.51	(1.03-2.2) **
**Relationship to the child**					
Other relative	5 (12)	10 (4)		Ref	
Mother	35 (81)	226 (91)	0.04	3.2	(1.0-10) **
Father	3 (7)	13 (5)	0.4	2.1	(0.4-11)
**Consider malaria vaccine safe**					
No	27 (63)	29 (12)		Ref	
Yes	16 (37)	220 (88)	<0.001	1.8	(1.4-2.3) **

** Significant variable at P < 0.05.

At the multivariable level, reduced willingness to vaccinate with the malaria vaccine was associated with prior experience of Adverse Events Following Immunization (AEFIs) (aPR 0.2, 95% CI: 0.05-0.5, p-value = 0.0013), having a relative other than the biological parent as the main decision maker (aPR 0.2,95% CI: 0.04-0.9, p-value = 0.04), and tertiary education (aPR = 0.14, 95%CI: 0.04-0.6, p-value = 0.007). Conversely, increased willingness to vaccinate was associated with prior receipt of malaria Intermittent preventive treatment in pregnancy (IPTp) (aPR = 6.2, 95%CI:1.6-23, p-value = 0.008) and confidence in vaccine safety (aPR = 13, 95%CI: 4.0-43, p-value = < 0.001) (Table 4). Perceived vaccine effectiveness was excluded from the multivariable model due to response overlap with perceived vaccine safety, as shown by identical response proportions (81%, Table 2). We retained perceived vaccine safety as it is a theoretically stronger determinant of vaccine uptake [23] (Table 4).

**Table 4:** Adjusted factors associated with willingness to vaccinate with the malaria vaccine among caregivers of U5 children at Masaka Regional Referral Hospital, Uganda, March 2025 (N = 292) [Multivariate analysis].

	Not willing to vaccinate	Willing to vaccinate						
**Characteristic**	**n (%)**	**n (%)**	**P-value**	**cPR**	**(95% CI)**	**P-value**	**aPR**	**(95% CI)**
**Caregiver age (Years)**								
15-24	19 (44)	93 (37)		Ref			Ref	
25-48	19 (44)	126 (51)	0.4	1.4	(0.7-2.7)	0.4	1.6	(0.6-4.7)
>48	5 (12)	30 (12)	0.7	1.2	(0.4-3.6)	0.2	3.0	(0.5-18)
**Level of Education**								
Primary	12 (28)	98 (39)		Ref			Ref	
Secondary	19 (44)	112 (45)	0.4	0.7	(0.33-1.5)	0.5	0.7	(0.2-2.0)
Tertiary/University	12 (28)	39 (16)	0.04	0.4	(0.16-0.96)	0.007	0.2	(0.04-0.6) **
**Consider the malaria vaccine effective**								
No	21 (49)	35 (14)		Ref			-	
Yes	22 (51)	214 (86)	<0.001	5.8	(2.9-12)			
**Child ever experienced AEFIᶲ**								
No	31 (72)	205 (82)		Ref			Ref	
Yes	12 (28)	44 (18)	0.1	0.6	(0.3-1.2)	0.0013	0.2	(0.05-0.5) **
**Received malaria IPTp⸸ (N=250)**								
No	8 (22)	11 (5)		Ref			Ref	
Yes	29 (78)	202 (95)	0.001	5.1	(1.9-14)	0.008	6.2	(1.6-23) **
**Consider malaria vaccine safe**								
No	27 (63)	29 (12)		Ref			Ref	
Yes	16 (37)	220 (88)	<0.001	1.8	(1.4-2.3)	< 0.001	13	(4.0-43) **
**Decision maker**								
Myself/ mother	26 (60)	142 (57)		Ref			Ref	
Spouse/ partner	11 (26)	98 (39)	0.2	1.6	(0.8-3.5)	0.1	2.3	(0.9-6.8)
Other family member	6 (14)	9 (4)	0.02	0.3	(0.09-0.8)	0.04	0.2	(0.04-0.9) **
**Relationship to the child**								
Other relative	5 (12)	10 (4)		Ref		-		
Mother	35 (81)	226 (91)	0.04	3.2	(1.0-10)			
Father	3 (7)	13 (5)	0.4	2.1	(0.4-11)			
**Knowledge about malaria spread**								
Not knowledgeable	1 (2)	27 (11)		Ref		-		
knowledgeable	23 (54)	66 (26)	0.03	0.1	(0.01-0.8) **			
Very knowledgeable	19 (44)	156 (63)	0.3	0.3	(0.04-2.4)			

**^⸸^IPTp:** Intermittent Preventive Treatment in pregnancy for malaria, **^ᶲ^ AEFIs**: Adverse Events Following Immunization ^**^ Represents significant findings at P < 0.05.

## Discussion

This study assessed caregivers’ awareness and willingness to vaccinate children under five years with the malaria vaccine at Masaka Regional Referral Hospital in south-central Uganda. We found low awareness of the malaria vaccine among caregivers but high willingness to vaccinate once informed. Factors associated with increased willingness were perceived vaccine safety and previous receipt of IPTp. Experience with AEFIs, non-biological parent main decision maker, and tertiary education were associated with reduced willingness to vaccinate.

### Awareness and willingness to vaccinate

Caregivers in this study demonstrated strong willingness to vaccinate their children under five years against malaria, with 85% expressing interest despite 58% reporting no prior awareness of the vaccine. This combination of low awareness and high willingness coincided with other studies;23.7% awareness with97.7% willingness in Nigeria, 12.3% with 90.6% in Burundi, 14.7% with 92.8% in Tanzania, and 62% with 91.4% willingness in a separate Ugandan cohort where reports of low baseline awareness alongside very high acceptance once caregivers were informed were observed [7,24–26] Similarly, a systematic review of 11 studies across sub-Saharan Africa reported an average caregiver acceptance rate of 82%, particularly in areas with high malaria transmission where perceived child vulnerability to malaria was associated with greater acceptance of preventive interventions [7]. The high willingness observed in our study may reflect caregivers’ personal or observed experiences with severe malaria and its associated treatment costs, motivating them to adopt long-term preventive measures such as vaccination [4,7]. These findings indicate strong community readiness for malaria vaccine rollout. Nonetheless, targeted health communication strategies remain essential to bridge existing knowledge gaps and further strengthen vaccine acceptance and uptake [26].

### Factors associated with caregivers’ increased willingness to vaccinate

Caregivers who had received a single dose of sulphadoxine/pyrimethamine (IPTp) during pregnancy were more likely to accept the malaria vaccine compared to those who had not. IPTp is delivered through antenatal care, and four or more ANC visits are the threshold at which most women receive it. While few studies directly examine the link between IPTp and vaccine acceptance, evidence shows that mothers who achieved the ANC threshold had good exposure to malaria preventive measures which fostered trust in the health system’s malaria interventions [1,27]. These findings suggest that ANC visits could be a plausible channel for malaria vaccine information ahead of delivery to strengthen early trust and promote vaccine uptake.

Caregivers who perceived the malaria vaccine as safe were more likely to vaccinate their children compared to those who did not, highlighting the role of trust in vaccine acceptance and uptake. These findings align with studies from Nigeria, where caregivers who perceived the vaccine as safe with benefits outweighing its risks were more willing to vaccinate their children [28,29]. Conversely, a meta-analysis across low- and middle-income countries reported low perceptions of vaccine safety among caregivers, which was associated with reluctance to vaccinate [30]. According to the Health Belief Model, health behaviors are shaped by individuals’ perceptions of risks, benefits, and barriers; thus, positive perceptions of vaccine safety could enhance willingness to vaccinate [31]. This underscores the need for targeted community health education campaigns using trusted communication channels to address vaccine safety rather than general vaccine promotion. Although perceived effectiveness was associated with vaccination willingness at bivariate level, it was not included into the multivariable model due to identical response proportions with perceived safety, suggesting collinearity. Instead, perceived safety was retained as it is theoretically a stronger vaccine uptake determinant compared to perceived effectiveness among determinants of health behavior in the health belief model [23,32].

### Factors associated with reduced willingness to vaccinate

Caregivers whose children had previously experienced AEFIs were less willing to vaccinate with the malaria vaccine compared to those without this experience, likely due to anxiety from recurrence and reduced trust in vaccine safety [9,33]. Our findings are similar to studies in Burundi and Nigeria, whereby individuals who had prior AEFIs experience were less likely to accept vaccination [9,26,28,34]. AEFIs are defined as unfavorable or unintended symptoms which follow immunization and do not necessarily have a causal relationship with the usage of the vaccine [35]. The AEFIs could range from mild reactions such as injection site erythema, swelling, pyrexia, and irritability to severe events such as febrile convulsions [36]. These findings highlight the need for the Ministry of Health to intensify community sensitization and health education on vaccine safety, using trusted channels such as radio, television, and community health workers. Additionally, targeted training for health workers to communicate accurately about AEFIs, alleviate caregiver fears, and support safe malaria vaccine rollout.

Caregivers with tertiary education were less willing to vaccinate their children compared to those with primary education. This may be linked to higher distrust of government, pharmaceutical firms, and healthcare systems among more educated caregivers, alongside exposure to misinformation from social media [37,38]. Similar findings were observed in other vaccination programs, including COVID-19, where higher education status was associated with lower vaccine acceptance due to distrust of the health care system, vaccine misconceptions, and fear of side effects [39–41]. In contrast, several studies reported those with a higher education as more willing to vaccinate their children than those with no formal education, an association linked to improved knowledge of vaccination benefits and positive attitudes towards child health [30,42,43]. This inconsistency suggests that education level alone does not determine willingness to vaccinate but instead shapes the kind of information caregivers would find credible. To address the hesitancy, health workers could integrate structured, dialogue-based social and behavior change communication into routine antenatal and child health contacts, tailored to concerns of more educated caregivers to build vaccine confidence.

Having relatives other than biological parents for main decision-making limited willingness to vaccinate. This may be due to reduced trust in the health system, compared to biological parents. Sensitization efforts should therefore deliberately reach extended family members and guardians, rather than assuming the biological parent is the primary decision maker.

### Policy implications and practical applications of study findings

ANC provides the first contact at which the malaria vaccine information is most likely to reach caregivers who are already engaged with the health system and predisposed to it. We recommend that malaria vaccine information be added to existing ANC counselling content rather than introduced as a separate campaign

Health workers require specific education guidance for caregivers with prior AEFI history and tertiary education. Caregivers with prior AEFI history should cover the distinction between common, self-limited reactions from rare severe events, whereas the tertiary-educated caregivers be given messages on vaccine provenance, regulatory approval and vaccine safety, which will be persuasive to vaccine uptake

### Strengths and limitations of the study

This study had several strengths. The study was conducted at Masaka Regional Referral Hospital, with a wide catchment area, making findings relevant to urban and peri-urban populations. Data collection took place one month prior to the national malaria vaccine rollout, providing timely evidence to inform policy and assess community awareness and readiness, as a pre-campaign baseline.

The study had several limitations. Social desirability bias may have arisen from interviewer-administered surveys in a healthcare setting, potentially inflating reported willingness to vaccinate. Additionally, recall bias may have affected reports of prior immunization experiences and child illnesses; to minimize this, questions were restricted to events occurring within six months prior to the interview. The study acknowledges potential response bias since willingness was measured after a brief study team orientation on the vaccine because prior awareness was too low to assess pre-orientation attitudes reliably at the required sample size. The 85% willingness may be an exaggeration from the actual community readiness, hence the associated factors should be interpreted within the post orientation context. Future studies should assess willingness after a considerable period of vaccine information provision to distinguish baseline attitudes from information-driven acceptance. The sample size was calculated based on prior estimated awareness of the malaria vaccine in a related study (18%) rather than the primary outcome of willingness to vaccinate, representing inconsistent methodology. However, since our study willingness (85%) exceeded the proportion used in the calculation, our study was adequately powered for primary analysis, and therefore future studies should base their sample size calculations on the primary outcome of interest. Selection bias since data was collected in an urban referral hospital setting, limiting generalizability to the rural population, who may differ in both awareness and channels through which they are reachable. In future, similar studies need to be conducted both at health facility and community levels to achieve adequate generalizability. Lastly, modified Poisson regression with standard errors was employed to minimize the risk of analytical bias arising from variance heterogeneity across comparison groups in the multivariable analysis.

## Conclusion

The majority of the participants were not aware of the malaria vaccine; however, they were willing to vaccinate their children when oriented. Prior experience of having a child with AEFIs, tertiary education and having other relative as decision maker were associated with reduced willingness to vaccinate, whereas having received malaria prophylaxis during pregnancy and trust in the safety of the malaria vaccine were associated with increased willingness to vaccinate. We recommended providing malaria vaccine information through antenatal care, prepare health workers to address the AEFI history and vaccine-safety concerns as distinct issues and prioritize safety-focused messaging over general awareness messaging where resources are limited.

## Abbreviations

AEFIs: Adverse Events Following Immunization
AE: Adverse Events
aPR: adjusted Prevalence Ratio
BeSD: Behavioral and Social Drivers of vaccination
cPR: Crude Prevalence Ratio
DHIS2: Data Health Information Software version 2
MCH: Maternal and Child Health
MRRH: Masaka Regional Referral Hospital
NRD: Non-Research Determination
U5: Children under five years of age
US CDC: United States Centers for Disease Control and Prevention
WHO: World Health Organization
