# Supplementary material for: Caregivers’ willingness and factors influencing uptake of the malaria vaccine among under-five children at Masaka Regional Referral Hospital, Uganda
Source: PLOS Glob Public Health. 2026 Oct 6;6(10):e0005764. doi: 10.1371/journal.pgph.0005764 (PMC13641375; doi:10.1371/journal.pgph.0005764)
Supplement: Supplementary file 1 [file pgph.0005764.s006.pdf]

No	Comment	Response
	**Reviewer 1**	
	**1. Critical factual inconsistency regarding vaccine awareness (Abstract vs. Results)**<br><br>There is a significant discrepancy between the abstract and the body of the manuscript regarding vaccine awareness. The abstract states: *"169 (58%) were aware of the malaria vaccine"*, while Table 2 and the Results section clearly show that 123 (42%) had heard about the malaria vaccine, and 169 (58%) had **not**. This represents an inversion of the finding in the abstract and is a factual error that must be corrected. As awareness is a secondary outcome of the study, its misreporting in the abstract materially misrepresents the study's findings.	The word "not" aware of the malaria vaccine was missed when reporting. However, this statement on vaccine awareness was omitted from the abstract as it was not the primary outcome of the study
	**2. Fundamental validity concern: "Orientation" provided prior to willingness assessment**<br><br>The study acknowledges in its limitations that vaccine information was provided to participants before their willingness was assessed. This is not merely a minor limitation — it fundamentally undermines the construct validity of the primary outcome measure. Willingness measured post-orientation reflects informed hypothetical acceptance rather than baseline community readiness. The 85% willingness figure, which is the headline finding, therefore cannot be interpreted as reflecting pre-existing attitudes in the population. This issue is acknowledged only briefly in the limitations (lines 310–312), but its implications are far-reaching: all regression associations identifying predictors of willingness are contingent on a post-orientation measure. The authors must discuss this more substantively, including how it affects the interpretation and generalizability of the adjusted prevalence	We thank the reviewer for this important observation. We have substantially expanded the discussion of this limitation and acknowledged that the achieved associations are contingent on a post orientation exposure. Please check lines 386-392

	ratios, and must be more cautious in the framing of their conclusions.	
	**3. Mismatch between sample size calculation and primary outcome**<br><br>The sample size calculation (N=226, inflated to 292) was based on an estimated proportion of caregivers *aware* of the malaria vaccine (18%), not on the primary outcome of willingness (85%). Using awareness as the basis for sample size when willingness is the primary analysis outcome is methodologically inconsistent. While the resulting sample size may have been sufficient for the willingness analysis, this discrepancy should be explicitly acknowledged and justified. If willingness was always the primary outcome, the sample size calculation should have been based on a relevant proportion for willingness.	This limitation has been acknowledged. Please check lines 392-397
	**4. Incomplete reporting of multivariable model construction (Table 3)**<br><br>Table 3 presents only five variables, yet the Methods section lists numerous independent variables assessed at the bivariate level (including marital status, place of residence, religion, number of children, comorbidities, distance from health centre, child history of malaria, religious/cultural beliefs, and others). Furthermore, the narrative in the Results section (lines 204–208) states that several variables were statistically significant at bivariate level — including being the main decision-maker, relationship to the child, knowledge of malaria spread, and discouragement by religious/cultural beliefs — yet none of these appear in Table 3, even in a reduced form. The authors must either: (a) include a complete bivariate analysis table showing all variables tested and their crude	A complete bivariate analysis table showing all variables tested and their crude prevalence ratios has been created. Please check Table 3.<br>In addition, Using backward elimination method, and after adjusting for confounders, we obtained associated factors as detailed in **Table 4**.

	prevalence ratios, or (b) clearly explain the variable selection process and why variables significant at the p<0.20 threshold were excluded from the multivariable model. The use of a stepwise forward selection approach is stated, but the criteria and steps are not transparent.	
	**5. The "consider malaria vaccine effective" variable: unexplained attenuation**<br><br>In Table 3, perceiving the malaria vaccine as effective is strongly associated with willingness at the bivariate level (cPR=1.45, p<0.001) but loses significance entirely in the multivariable model (aPR=1.2, p=0.23). This likely reflects collinearity between perceived effectiveness and perceived safety (both assessed with the same 236/292 = 81% positive response). The authors should explicitly acknowledge and discuss this collinearity, as it affects the interpretation of both variables in the final model.	Perceived vaccine effectiveness was excluded from the multivariable model due to response overlap with perceived vaccine safety, as shown by identical response proportions ([81%] in Table 2). We retained perceived vaccine safety as it is theoretically stronger regarding vaccine uptake among beneficiaries. This explanation has been given both in the methods and the discussion section
	**6. Borderline statistical significance of the IPTp association**<br><br>The adjusted prevalence ratio for IPTp receipt (aPR=1.45, 95%CI 1.00–2.10, p=0.047) shows a lower confidence bound at exactly 1.00, indicating borderline statistical significance. This finding should be interpreted with appropriate caution. The authors should note that this borderline result, combined with the small number of IPTp-negative participants (n=8 not willing; n=11 willing), limits confidence in this association. The claim in the Discussion that IPTp receipt is a meaningful predictor of willingness should be tempered accordingly	We thank the reviewer for raising this concern. Following re-analysis of the data using backward elimination while controlling for potential confounders, the association between IPTp receipt and willingness to vaccinate was strengthened considerably. The revised estimate provides stronger statistical evidence for this association.
.	**7. Ethics approval: absence of Ugandan institutional ethics review**	The Ugandan ethics review was not sought since the study was not considered research. NRD approved by the US CDC, and other documents

	The study reports obtaining a Non-Research Determination (NRD) from the US CDC, which classifies the activity as public health surveillance/practice rather than research under US federal regulations, and administrative clearance from the hospital. However, no mention is made of review by a Ugandan institutional ethics board (e.g., Makerere University School of Public Health Research Ethics Committee or the Uganda National Council for Science and Technology). Publication of data from human participants in a peer-reviewed journal typically requires independent ethics review, even for public health programme evaluations. The authors should clarify whether Ugandan ethics review was sought and either provide the relevant approval reference or justify the adequacy of the NRD pathway under Ugandan national regulations.	including the Ministry of Health Uganda Directive document, the Umbrella protocol project determination document were attached in the submission portal. These documents provide permission for individual data collection from communities for public health surveillance/ practice.
	**The SBCC tool: unexplained and unreferenced**<br><br>In the Strengths section (lines 300–301), the authors state they used "a standardized and validated data collection tool (the SBCC tool)." This is the only mention of the SBCC (Social and Behaviour Change Communication) tool in the entire manuscript. It is not described in the Methods, and no reference or citation is provided for its validation. Either this tool should be properly described and cited in the Methods section, or the claim of standardisation and validation should be removed if it refers simply to the semi-structured questionnaire developed for this study.	This sentence was omitted from the manuscript. Since most of the questions were adopted from the Behavioural and Social Drivers of vaccination tool (BeSD). The study strengths section was adjusted appropriately.
	**9. Abbreviations list includes unused terms**<br><br>The list of abbreviations includes "aOR: adjusted Odds Ratio" and "cOR: Crude Odds Ratio." These abbreviations are never used in the manuscript; the analysis correctly uses	The abbreviations list has been adjusted appropriately

	prevalence ratios (aPR, cPR). These unused abbreviations should be removed to avoid confusion.	
	**10. Inconsistent and imprecise mortality data in the Background**<br><br>Line 89 states that "mortality of U5s ranging from 60-50's per 1000 population in the last 5 years (2020-2024)." This phrasing is grammatically awkward and numerically unclear (the range "60-50's" appears to be a typographical error or an unconventional way of expressing declining rates). This should be revised to clearly present the mortality trend with proper units and formatting.	Malaria mortality data for Masaka RRH were depicted from DHIS2. The report has been adjusted appropriately. Lines: 83 and 84
	**11. Duplicate reference list**<br><br>The manuscript (revised version with track changes) contains two complete and duplicated reference lists starting at lines 363 and 433, respectively. While the response letter states that duplicates have been removed, they remain visible in the submitted document. This must be corrected in the final version.	Duplicates have been removed from the reference list
	**12. Reference quality concerns**<br><br>Several references present quality issues:<br><br>• Reference 3 (Peter Omms Oumo PT et al., 2022) is formatted in a non-standard way and appears to be an unpublished or grey literature document with no journal, DOI, or verifiable source. If this is a report, it should be cited with full institutional details.<br>• Reference 12 ("consortium M. Malaria drops to no.2...") is also grey literature with an incomplete and informal citation. This should be either formally cited with	These references have been corrected in the reference list.<br><br>Former reference 12 by Malaria consortium has been omitted and replaced by more updated literature

	full source details or replaced with a peer-reviewed reference.<br>• Reference 26 ("hub R. Health Belief Model. 2025") and Reference 28 ("CDC. Adverse Events Following Immunization (AEFI). 2025") should include full URLs and access dates as they are web-based sources.	
	**13. Causal language in a cross-sectional design**<br><br>The Discussion uses language that occasionally implies directionality or causality (e.g., "reduced trust in vaccine safety," "may stem from anxiety about repeating similar adverse events"). While such hypothesised mechanisms are appropriate for discussion, the authors should consistently qualify statements with language appropriate to a cross-sectional design (e.g., "associated with," "observed alongside").	The discussion section has been adjusted appropriately to cross-sectional design language, to demonstrate associations. Please check the discussion section.
	**14. IPTp restricted to female participants — insufficient clarification in text**<br><br>The IPTp analysis was restricted to the 250 female participants, which is appropriate and now noted in Table 3 (N=250). However, the main Results text does not adequately signal this sub-group restriction. Readers may be confused by the change in denominator. A brief clarifying sentence in the Results text would be helpful.	The results section has been updated to outline that IPTp only applied to females in the study, who summed up to 250 people
	**15. Discussion of tertiary education finding: speculative causal mechanism**<br><br>The proposed mechanism for lower willingness among tertiary-educated caregivers — that they are more susceptible to misinformation due to	This discussion section of tertiary education has been corrected appropriately. Please check lines: 359-371.

	stronger research skills (lines 282–285) — is speculative and potentially unflattering to a specific demographic without strong empirical grounding. The reference to the MMR-autism controversy (reference 30) as an example is not directly relevant to malaria vaccination in Uganda. The authors have added additional references (31–33), which is welcome, but the interpretation should be presented more cautiously, acknowledging competing explanations (e.g., heightened risk perception, access to contradictory information, or greater scrutiny of pharmaceutical interventions).	
	**16. Data availability and PLOS policy**<br><br>PLOS requires that all data underlying the findings be made freely available. The authors cite confidentiality restrictions and refer requesters to an institutional email address. While PLOS does allow exceptions, the justification provided is minimal. The authors should more explicitly state the nature of the confidentiality concern (e.g., personal identifying information) and clarify whether an anonymised or de-identified dataset could be made available, consistent with PLOS data availability policy.	We confirm that a de-identified and anonymized dataset with all direct and indirect identifiers removed can be made available to researchers upon request subject to approval by the Uganda National Institute of Public Health and the US CDC which provided oversight for this study. We have revised the data availability statement
	**17. Title includes the study date "March 2025"**<br><br>Including the specific month and year in the title is unusual for a research article and may reduce the long-term discoverability and readability of the paper. The authors should consider whether this specificity adds value to the title or whether it would be better addressed within the Methods section.	The title has been adjusted and excluded “March 2025”. These specifics of month and year have been maintained in the subtitles within the manuscript body.
	**REVIEWER 2**	

	It would strengthen the manuscript to present a **theoretical or conceptual model** in the introduction or literature review. A diagram illustrating the relationships between key variables could help guide the interpretation of the findings, clarify causal pathways, and support the identification of potential **confounding factors**. Such a framework would also assist in justifying variable selection and analytical choices.	A conceptual framework has been included in the manuscript as part of the background section, Fig 1
	Similarly, the **inclusion and exclusion criteria** could be described in greater detail to enhance transparency and reproducibility, and to allow readers to better assess potential selection bias.	The inclusion and exclusion criteria have been detailed under the methods section. Lines: 151-156
	The issue of **missing data** requires further clarification. The authors could indicate:<br>• Whether missing data were present, and for which variables<br>• The extent and patterns<br>• The methods used to handle missing data<br>• The potential influence of missing data on the study outcomes and conclusions.	Missing data were present in a small number of variables like religious and cultural beliefs where participants declined to respond, and were assessed to be low at <2%. More details are included in the manuscript, Lines 226-231
	From an analytical perspective, the authors could report whether there is an **estimate of variability within each group**, and whether variance is comparable across groups.	We acknowledge that there could have been variance across groups. However, robust standard errors in the modified Poisson regression model were used to account for potential variance across comparison groups, ensuring valid prevalence ratio estimates regardless of variance differences between groups.

	In addition, the role of **social determinants of health** warrants deeper exploration. Specifically:<br><br>• Are there observable differences between socio-economic groups (e.g., poorest vs. wealthiest quintiles, geographic disparities) (is the willingness and awareness different, taking into account the geographic disparities?<br>• How were social inequalities and disparities incorporated into the analysis?<br>• What implications do these disparities have for the interpretation of the findings?	This study did not explicitly examine socioeconomic inequalities or geographic disparities in malaria vaccine willingness. Similarly, formal wealth quintile analysis and geographic mapping were not done in this study<br>However, several variables captured in this study served as proxies for socioeconomic status and geographic disparity, such as residence (urban Versus (V) rural, distance to the health centre, education level, accessing childhood vaccinations were collected and examined as independent variables. These variables provided a partial picture of socio economic and geographic disparities within the study population
	The authors could more explicitly address **potential sources of bias** that could influence the direction or magnitude of the results. This includes, but is not limited to:<br><br>• Selection bias;<br>• Design-related bias;<br>• Measurement or instrumentation bias;<br>• Analytical bias.<br><br>The manuscript could describe the strategies used to **minimize or control bias**	The potential sources of bias and control strategies have been explained under study limitations
	The manuscript could describe<br><br>How major **confounding variables** were identified and managed.	Potential confounding variables were identified a priori based on existing literature especially using the health belief model in the background. (Lines: 96-123)<br>During analysis all values with P<0.2 were included in the multivariable modified Poisson regression model and using backward elimination, the independent effect of each variable was estimated while adjusting for the others. In this way, confounders were managed.
	Regarding the methodology, what justifies the Poisson regression and the systematic	Modified Poisson regression was used because of the high prevalence

	sampling? Did you have a sampling frame or a list?	of willingness to vaccinate-at 85%. (Lines 216-219)<br>The attendance list of caregivers whose children were present for the day's immunization was created and used as the sampling frame. (Lines: 168-173)
	Were previous studies similar to this study, published in the scientific literature?<br>• What were the major findings?<br>• How is your study similar to or different from those studies?<br><br>What are the policy implications of your study? Could you integrate this aspect into the discussion section?	This has been addressed in the discussion section of the manuscript
