# Supplementary material for: Caregivers’ willingness and factors influencing uptake of the malaria vaccine among under-five children at Masaka Regional Referral Hospital, Uganda
Source: PLOS Glob Public Health. 2026 Oct 6;6(10):e0005764. doi: 10.1371/journal.pgph.0005764 (PMC13641375; doi:10.1371/journal.pgph.0005764)
Supplement: Supplementary file 2 [file pgph.0005764.s007.pdf]

No	Comment	Response
1	The discussion section needs some improvement: specificially, could you add some sub-headings to organize the discussion? Could you add a sub headings regarding the following: policy implications and practical application of the findings; a subheading regarding "strength and limitations of the study" or other headings you and the research team judge that can be useful.	The discussion section has been revised. Subheadings regarding policy implications and practical application of findings, strength and limitations of the study and other subheadings have been added
2	The language of the document requires a comprehensive review. Professional proofreading and editing of the entire document are strongly recommended. Furthermore, a significant structural revision is necessary to enhance overall flow and coherence.	The document has been proofread and edited to enhance coherence
