# Supplementary material for: Caregivers’ willingness and factors influencing uptake of the malaria vaccine among under-five children at Masaka Regional Referral Hospital, Uganda
Source: PLOS Glob Public Health. 2026 Oct 6;6(10):e0005764. doi: 10.1371/journal.pgph.0005764 (PMC13641375; doi:10.1371/journal.pgph.0005764)
Supplement: Supplementary file 3 [file pgph.0005764.s002.docx]

**Reviewer Recommendations and Comments**

Reviewer:

LAST NAME: DEMES

FIRST NAME: JOSEPH ADRIEN EMMANUEL

**Title of article:**

Caregivers’ Willingness and Factors Influencing Uptake of the Malaria Vaccine Among Under-Five Children at Masaka Regional Referral Hospital, Uganda, March 2025

**Major comments:**

The authors should be commended for focusing on an understudied topic: Caregivers’ Willingness and Factors Influencing Uptake of the Malaria Vaccine Among Under-Five Children.

It would strengthen the manuscript to present a **theoretical or conceptual model** in the introduction or literature review. A diagram illustrating the relationships between key variables could help guide the interpretation of the findings, clarify causal pathways, and support the identification of potential **confounding factors**. Such a framework would also assist in justifying variable selection and analytical choices.

Similarly, the **inclusion and exclusion criteria** coould be described in greater detail to enhance transparency and reproducibility, and to allow readers to better assess potential selection bias.

The issue of **missing data** requires further clarification. The authors could indicate:

- Whether missing data were present, and for which variables
- The extent and patterns
- The methods used to handle missing data
- The potential influence of missing data on the study outcomes and conclusions.

From an analytical perspective, the authors could report whether there is an **estimate of variability within each group**, and whether variance is comparable across groups.

In addition, the role of **social determinants of health** warrants deeper exploration. Specifically:

- Are there observable differences between socio-economic groups (e.g., poorest vs. wealthiest quintiles, geographic disparities) (is the willingness and awareness different, taking into account the geographic disparities?
- How were social inequalities and disparities incorporated into the analysis?
- What implications do these disparities have for the interpretation of the findings?

Finally, the authors could more explicitly address **potential sources of bias** that could influence the direction or magnitude of the results. This includes, but is not limited to:

- Selection bias;
- Design-related bias;
- Measurement or instrumentation bias;
- Analytical bias.

The manuscript could describe the strategies used to **minimize or control bias**, including how major **confounding variables** were identified and managed.

It could be interesting to find a way to make it easy for the readers to understand the paper coherently.

What do you think?

Regarding the methodology, what justifies the Poisson regression and the systematic sampling? Did you have a sampling frame or a list?

Also, review the whole document structure and flow, and coherence so that everything could have a thread from the beginning to the end.

**Minor comments:**

The language of the document requires a comprehensive review. Professional proofreading and editing of the entire document are strongly recommended. Furthermore, a significant structural revision is necessary to enhance overall flow and coherence.

Were previous studies similar to this study, published in the scientific literature?

- What were the major findings?
- How is your study similar to or different from those studies?

What are the policy implications of your study? Could you integrate this aspect into the discussion section?

**Decision:** A minor revision is needed before publication.
