# Supplementary material for: Caregivers’ willingness and factors influencing uptake of the malaria vaccine among under-five children at Masaka Regional Referral Hospital, Uganda
Source: PLOS Glob Public Health. 2026 Oct 6;6(10):e0005764. doi: 10.1371/journal.pgph.0005764 (PMC13641375; doi:10.1371/journal.pgph.0005764)
Supplement: Supplementary file 4 [file pgph.0005764.s003.docx]

**PEER REVIEW — PLOS Global Public Health**

**Manuscript ID:** PGPH-D-25-03668 R1 **Title:** *Caregivers' Willingness and Factors Influencing Uptake of the Malaria Vaccine Among Under-Five Children at Masaka Regional Referral Hospital, Uganda, March 2025*

**Summary assessment**

This revised manuscript addresses an important and timely public health question: understanding the determinants of caregiver willingness to vaccinate children under five with the newly introduced R21 malaria vaccine in Uganda, a country carrying one of the highest global malaria burdens. The study was conducted immediately prior to Uganda's national vaccine rollout in April 2025, which lends it considerable policy relevance. The authors have made a commendable effort to address several of the previous reviewers' concerns. However, a number of substantive methodological, analytical, and reporting issues remain that require revision before the manuscript can be considered for publication.

**Major concerns**

**1. Critical factual inconsistency regarding vaccine awareness (Abstract vs. Results)**

There is a significant discrepancy between the abstract and the body of the manuscript regarding vaccine awareness. The abstract states: *"169 (58%) were aware of the malaria vaccine"*, while Table 2 and the Results section clearly show that 123 (42%) had heard about the malaria vaccine, and 169 (58%) had **not**. This represents an inversion of the finding in the abstract and is a factual error that must be corrected. As awareness is a secondary outcome of the study, its misreporting in the abstract materially misrepresents the study's findings.

**2. Fundamental validity concern: "Orientation" provided prior to willingness assessment**

The study acknowledges in its limitations that vaccine information was provided to participants before their willingness was assessed. This is not merely a minor limitation — it fundamentally undermines the construct validity of the primary outcome measure. Willingness measured post-orientation reflects informed hypothetical acceptance rather than baseline community readiness. The 85% willingness figure, which is the headline finding, therefore cannot be interpreted as reflecting pre-existing attitudes in the population. This issue is acknowledged only briefly in the limitations (lines 310–312), but its implications are far-reaching: all regression associations identifying predictors of willingness are contingent on a post-orientation measure. The authors must discuss this more substantively, including how it affects the interpretation and generalizability of the adjusted prevalence ratios, and must be more cautious in the framing of their conclusions.

**3. Mismatch between sample size calculation and primary outcome**

The sample size calculation (N=226, inflated to 292) was based on an estimated proportion of caregivers *aware* of the malaria vaccine (18%), not on the primary outcome of willingness (85%). Using awareness as the basis for sample size when willingness is the primary analysis outcome is methodologically inconsistent. While the resulting sample size may have been sufficient for the willingness analysis, this discrepancy should be explicitly acknowledged and justified. If willingness was always the primary outcome, the sample size calculation should have been based on a relevant proportion for willingness.

**4. Incomplete reporting of multivariable model construction (Table 3)**

Table 3 presents only five variables, yet the Methods section lists numerous independent variables assessed at the bivariate level (including marital status, place of residence, religion, number of children, comorbidities, distance from health centre, child history of malaria, religious/cultural beliefs, and others). Furthermore, the narrative in the Results section (lines 204–208) states that several variables were statistically significant at bivariate level — including being the main decision-maker, relationship to the child, knowledge of malaria spread, and discouragement by religious/cultural beliefs — yet none of these appear in Table 3, even in a reduced form. The authors must either: (a) include a complete bivariate analysis table showing all variables tested and their crude prevalence ratios, or (b) clearly explain the variable selection process and why variables significant at the p<0.20 threshold were excluded from the multivariable model. The use of a stepwise forward selection approach is stated, but the criteria and steps are not transparent.

**5. The "consider malaria vaccine effective" variable: unexplained attenuation**

In Table 3, perceiving the malaria vaccine as effective is strongly associated with willingness at the bivariate level (cPR=1.45, p<0.001) but loses significance entirely in the multivariable model (aPR=1.2, p=0.23). This likely reflects collinearity between perceived effectiveness and perceived safety (both assessed with the same 236/292 = 81% positive response). The authors should explicitly acknowledge and discuss this collinearity, as it affects the interpretation of both variables in the final model.

**6. Borderline statistical significance of the IPTp association**

The adjusted prevalence ratio for IPTp receipt (aPR=1.45, 95%CI 1.00–2.10, p=0.047) shows a lower confidence bound at exactly 1.00, indicating borderline statistical significance. This finding should be interpreted with appropriate caution. The authors should note that this borderline result, combined with the small number of IPTp-negative participants (n=8 not willing; n=11 willing), limits confidence in this association. The claim in the Discussion that IPTp receipt is a meaningful predictor of willingness should be tempered accordingly.

**7. Ethics approval: absence of Ugandan institutional ethics review**

The study reports obtaining a Non-Research Determination (NRD) from the US CDC, which classifies the activity as public health surveillance/practice rather than research under US federal regulations, and administrative clearance from the hospital. However, no mention is made of review by a Ugandan institutional ethics board (e.g., Makerere University School of Public Health Research Ethics Committee or the Uganda National Council for Science and Technology). Publication of data from human participants in a peer-reviewed journal typically requires independent ethics review, even for public health programme evaluations. The authors should clarify whether Ugandan ethics review was sought and either provide the relevant approval reference or justify the adequacy of the NRD pathway under Ugandan national regulations.

**MINOR CONCERNS**

**8. The SBCC tool: unexplained and unreferenced**

In the Strengths section (lines 300–301), the authors state they used "a standardized and validated data collection tool (the SBCC tool)." This is the only mention of the SBCC (Social and Behaviour Change Communication) tool in the entire manuscript. It is not described in the Methods, and no reference or citation is provided for its validation. Either this tool should be properly described and cited in the Methods section, or the claim of standardisation and validation should be removed if it refers simply to the semi-structured questionnaire developed for this study.

**9. Abbreviations list includes unused terms**

The list of abbreviations includes "aOR: adjusted Odds Ratio" and "cOR: Crude Odds Ratio." These abbreviations are never used in the manuscript; the analysis correctly uses prevalence ratios (aPR, cPR). These unused abbreviations should be removed to avoid confusion.

**10. Inconsistent and imprecise mortality data in the Background**

Line 89 states that "mortality of U5s ranging from 60-50's per 1000 population in the last 5 years (2020-2024)." This phrasing is grammatically awkward and numerically unclear (the range "60-50's" appears to be a typographical error or an unconventional way of expressing declining rates). This should be revised to clearly present the mortality trend with proper units and formatting.

**11. Duplicate reference list**

The manuscript (revised version with track changes) contains two complete and duplicated reference lists starting at lines 363 and 433, respectively. While the response letter states that duplicates have been removed, they remain visible in the submitted document. This must be corrected in the final version.

**12. Reference quality concerns**

Several references present quality issues:

- Reference 3 (Peter Omms Oumo PT et al., 2022) is formatted in a non-standard way and appears to be an unpublished or grey literature document with no journal, DOI, or verifiable source. If this is a report, it should be cited with full institutional details.
- Reference 12 ("consortium M. Malaria drops to no.2...") is also grey literature with an incomplete and informal citation. This should be either formally cited with full source details or replaced with a peer-reviewed reference.
- Reference 26 ("hub R. Health Belief Model. 2025") and Reference 28 ("CDC. Adverse Events Following Immunization (AEFI). 2025") should include full URLs and access dates as they are web-based sources.

**13. Causal language in a cross-sectional design**

The Discussion uses language that occasionally implies directionality or causality (e.g., "reduced trust in vaccine safety," "may stem from anxiety about repeating similar adverse events"). While such hypothesised mechanisms are appropriate for discussion, the authors should consistently qualify statements with language appropriate to a cross-sectional design (e.g., "associated with," "observed alongside").

**14. IPTp restricted to female participants — insufficient clarification in text**

The IPTp analysis was restricted to the 250 female participants, which is appropriate and now noted in Table 3 (N=250). However, the main Results text does not adequately signal this sub-group restriction. Readers may be confused by the change in denominator. A brief clarifying sentence in the Results text would be helpful.

**15. Discussion of tertiary education finding: speculative causal mechanism**

The proposed mechanism for lower willingness among tertiary-educated caregivers — that they are more susceptible to misinformation due to stronger research skills (lines 282–285) — is speculative and potentially unflattering to a specific demographic without strong empirical grounding. The reference to the MMR-autism controversy (reference 30) as an example is not directly relevant to malaria vaccination in Uganda. The authors have added additional references (31–33), which is welcome, but the interpretation should be presented more cautiously, acknowledging competing explanations (e.g., heightened risk perception, access to contradictory information, or greater scrutiny of pharmaceutical interventions).

**16. Data availability and PLOS policy**

PLOS requires that all data underlying the findings be made freely available. The authors cite confidentiality restrictions and refer requesters to an institutional email address. While PLOS does allow exceptions, the justification provided is minimal. The authors should more explicitly state the nature of the confidentiality concern (e.g., personal identifying information) and clarify whether an anonymised or de-identified dataset could be made available, consistent with PLOS data availability policy.

**17. Title includes the study date "March 2025"**

Including the specific month and year in the title is unusual for a research article and may reduce the long-term discoverability and readability of the paper. The authors should consider whether this specificity adds value to the title or whether it would be better addressed within the Methods section.

**ASSESSMENT OF PLOS GLOBAL PUBLIC HEALTH CRITERIA**

| **Criterion** | **Assessment** |
| --- | --- |
| **Global public health relevance** | Strong - malaria vaccine rollout in a high-burden country is a clear global health priority |
| **Originality** | Moderate - similar studies exist across sub-Saharan Africa; the main added value is the pre-rollout timing and Ugandan context |
| **Methodological rigor** | Requires revision - concerns about primary outcome validity, sample size justification, and incomplete reporting of the analytical model |
| **Clarity and completeness of reporting** | Requires revision - abstract error, incomplete variable reporting, duplicate references |
| **Ethics** | Requires clarification - Ugandan ethics review not mentioned |
| **Data availability** | Borderline - justification for restricted access needs strengthening |

**Recommendation**

**Major Revision Required.**

The study addresses a genuinely important and timely public health question, and several of the previous reviewers' concerns have been addressed. However, the factual error in the abstract, the validity concerns surrounding the post-orientation willingness measure, the incomplete reporting of the analytical model, and the unresolved ethics approval question represent issues that must be adequately addressed before this manuscript can be considered for publication. I encourage the authors to engage thoroughly with each point raised and to revise accordingly.
