# Supplementary material for: Caregivers’ willingness and factors influencing uptake of the malaria vaccine among under-five children at Masaka Regional Referral Hospital, Uganda
Source: PLOS Glob Public Health. 2026 Oct 6;6(10):e0005764. doi: 10.1371/journal.pgph.0005764 (PMC13641375; doi:10.1371/journal.pgph.0005764)
Supplement: Supplementary file 5 [file pgph.0005764.s004.docx]

**POINT-BY-POINT RESPONSE TO REVIEWERS’ COMMENTS**

| **No** | **Comment** | **Response** |
| --- | --- | --- |
|  | **Editor** |  |
| **1** | Please provide a detailed online Financial Disclosure statement. This is published with the article. It must therefore be completed in full sentences and contain the exact wording you wish to be published.  a) State the initials, alongside each funding source, of each author to receive each grant. For example: “This work was supported by the National Institutes of Health (####### to AM; ###### to CJ) and the National Science Foundation (###### to AM).”  b) State what role the funders took in the study. If the funders had no role in your study, please state: “The funders had no role in study design, data collection and analysis, decision to publish, or preparation of the manuscript.” | An online Financial Disclosure statement has been provided  Initials have been indicated alongside the funding source for all authors who received the grant. It was one grant from US CDC but received through Makerere University School of Public Health and the Uganda Public Health Fellowship Program, hence one grant number documented.  A statement of “The funders had no role in study design, data collection, and analysis, decision to publish or preparation of the manuscript” has been added. |
| **2** | Please update your online Competing Interests statement. If you have no competing interests to declare, please state: “The authors have declared that no competing interests exist.” | A statement that “ The authors have declared that no competing interests exist” has been added |
| **3** | Please also provide non-author contact information (phone/email/hyperlink) for a data access committee, ethics committee, or other institutional body to which data requests may be sent. If no institutional body is available to respond to requests for your minimal data, please consider if there any institutional representatives who did not collaborate in the study, and are not listed as authors on the manuscript, who would be able to hold the data and respond to external requests for data access? If so, please provide their contact information (i.e., email address). Please also provide details on how you will ensure persistent or long-term data storage and availability. | The datasets upon which our findings are based belong to the Uganda Public Health Fellowship Program. For confidentiality reasons, the datasets are not publicly available. The datasets can be made available upon reasonable request from the responsible officer with permission from the Uganda Public Health Fellowship Program. Request can be directed at: [it@uniph.go.ug](mailto:it@uniph.go.ug) |
| **4** | Please ensure that you refer to Tables 1, 2 and 3 in your text as, if accepted, production will need these references to link the reader to the tables. | We have indicated table names at the end of text describing the table for easy referencing |
| **5** | If the reviewer comments include a recommendation to cite specific previously published works, please review and evaluate these publications to determine whether they are relevant and should be cited. There is no requirement to cite these works unless the editor has indicated otherwise. | Previously published work was cited during the discussion section as advised by the reviewers. |
| **6** | Please review your reference list to ensure that it is complete and correct. If you have cited papers that have been retracted, please include the rationale for doing so in the manuscript text, or remove these references and replace them with relevant current references. Any changes to the reference list should be mentioned in the rebuttal letter that accompanies your revised manuscript. If you need to cite a retracted article, indicate the article’s retracted status in the References list and also include a citation and full reference for the retraction notice. | We have reviewed the reference list and ensured its complete and correct. The references were duplicated earlier and we have removed the duplicates. In addition, we have added links to some references that were web pages, to enable readers easily access these websites. |
|  | **Reviewer 1** |  |
| 7 | Do you think your current definition of a caregiver as contained in line 110 to 111, and which I assume is somewhat of your inclusion criteria, is encompassing enough to cover those participants in your study who are not primary caregivers or main decision maker for the children? You might want to modify your definition accordingly. | We thank you for raising this important comment. We have refocused the definition of the caregiver to make it more sensitive and accommodate more participants in the study. This was modified from a definition of a caregiver from another study cited. Please check lines 109-111 for details. |
| 8 | I think reporting your percentages to the nearest one decimal place (e.g. 5.4%) will help ensure consistency in your reporting. For instance, percentage of children who sleep or do not sleep under a mosquito net do not add up to 100, because 'yes' was rounded up to a whole number while 'no' was rounded to one decimal place. I believe the percentage of 'Mother' as main decision maker is incorrect. It is currently listed as 5.8%. | We have rounded off the percentages to the nearest decimal place especially in Table 1 and across all the other table, ensuring each variable adds up to 100%. We have adjusted the percentage of ‘mother’ as main decision maker. Please check table 1. |
| 9 | In line 184, you mention that majority of caregiver say they could buy the malaria vaccine if it were available for sale. I wonder if you gave them an estimated cost per dose and how many does they will need to get before they responded to this question. | We have adjusted the statement on the participant’s willingness to buy the vaccine if it were sold by including the likely prices for each dose incase it were to be sold in public health facilities. This price range had been mentioned to the respondents during data collection. Please see lines 186-187 for details. |
| 10 | Your Table 2 is empty | We have adjusted and reinstituted Table 2. Please check page 9 for the inclusion. |
|  | **Reviewer 2** |  |
| 11 | The manuscript should more explicitly discuss potential biases introduced by providing information (“orientation”) about the vaccine during data collection, which may have influenced willingness responses and introduced potential response bias. | Potential biases, such as response bias have been added to the limitations. Please check lines 310-312 for details |
| 12 | Variable denominators across some analyses suggest missing data or eligibility differences. The denominator (n) for each group appears to change from variable to variable, for example, in Table 3, IPTp analyses are based on 37 participants rather than 43 in the “not willing to vaccinate” group, and on 213 rather than 247 in the “willing to vaccinate” group. Clarification or justification should be provided. | Thank you for the concern. We have observed the denominators for each group in table 3 and adjusted where needed. IPTp analyses were done among 250 participants who were the females in the study. Among these, 37 were not willing to vaccinate with the malaria vaccine while 213 were willing to vaccinate. We have included the specific N as 250 for the variable-‘received malaria IPTp”. Please check Table 3 for details. |
| 13 | Some findings, particularly the lower willingness among caregivers with tertiary education, seem counterintuitive and would benefit from deeper interpretation or hypothesized mechanisms grounded in existing literature. Only one reference was provided to support the strength of this claim. Perhaps digging more into existing literature would provide a better standing for this finding. | The finding on lower willingness among caregivers with tertiary education has been further discussed to point out likely causes and appropriate references have been added. Please check lines 282-290 for details |
| 14 | As data were collected in a referral hospital setting, concerns with generalizability to rural or community-based populations should be more clearly acknowledged | This is an important observation by the reviewer. We have incorporated it under limitations and provided a recommendation for future research. Please check lines 312-315 for details |
| 15 | Some clarification is needed regarding the measurement of adverse events following immunization (AEFI), specifically whether these were caregiver-reported or clinically verified, and whether severity was assessed | AEFIs were caregiver-reported and not clinically verified, and severity was not assessed. This has been clarified in the text. Please check lines 142-144 for details |
| 16 | References are duplicated from line 409 | We have taken note of this important comment and deleted the duplicate references from the reference list. Please check page 15-17 for details |
