# Supplementary material for: Caregivers’ willingness and factors influencing uptake of the malaria vaccine among under-five children at Masaka Regional Referral Hospital, Uganda
Source: PLOS Glob Public Health. 2026 Oct 6;6(10):e0005764. doi: 10.1371/journal.pgph.0005764 (PMC13641375; doi:10.1371/journal.pgph.0005764)
Supplement: Supplementary file 6 [file pgph.0005764.s005.docx]

**Reviewer Recommendations and Comments**

Reviewer:

LAST NAME: DEMES

FIRST NAME: JOSEPH ADRIEN EMMANUEL

**Title of article:**

Caregivers’ Willingness and Factors Influencing Uptake of the Malaria Vaccine Among Under-Five Children at Masaka Regional Referral Hospital, Uganda, March 2025

**Major comments:**

The authors should be commended for focusing on an understudied topic: Caregivers’ Willingness and Factors Influencing Uptake of the Malaria Vaccine Among Under-Five Children.

This is a great improvement. This version has a theoretical model that guides the study.

The discussion section needs some improvement: specificially, could you add some sub-headings to organize the discussion? Could you add a sub headings regarding the following: policy implications and practical application of the findings; a subheading regarding “strength and limitations of the study” or other headings you and the research team judge that can be useful.

What do you think?

Also, review the whole document structure and flow, and coherence so that everything could have a thread from the beginning to the end.

**Minor comments:**

The language of the document requires a comprehensive review. Professional proofreading and editing of the entire document are strongly recommended. Furthermore, a significant structural revision is necessary to enhance overall flow and coherence.

What are the policy implications of your study? Could you integrate this aspect into the discussion section?

**Decision:** A minor revision is needed before publication.
