# Supplementary material for: Caregivers’ willingness and factors influencing uptake of the malaria vaccine among under-five children at Masaka Regional Referral Hospital, Uganda
Source: PLOS Glob Public Health. 2026 Oct 6;6(10):e0005764. doi: 10.1371/journal.pgph.0005764 (PMC13641375; doi:10.1371/journal.pgph.0005764)
Supplement: Peer review history

# Peer review: Decision Letter 0

**DOI:** 10.1371/journal.pgph.0005764.r001

**Authors:** Collins Asweto

7 Jan 2026

PGPH-D-25-03668

Caregivers’ Willingness and Factors Influencing Uptake of the Malaria Vaccine Among Under-Five Children at Masaka Regional Referral Hospital, Uganda, March 2025

PLOS Global Public Health

Dear Annet,

Thank you for submitting your manuscript to PLOS Global Public Health. After careful consideration, we feel that it has merit but does not fully meet PLOS Global Public Health’s publication criteria as it currently stands. Therefore, we invite you to submit a revised version of the manuscript that addresses the points raised during the review process.

Please submit your revised manuscript by 2nd March 2026. If you will need more time than this to complete your revisions, please reply to this message or contact the journal office at globalpubhealth@plos.org. When you're ready to submit your revision, log on to https://www.editorialmanager.com/pgph/ and select the 'Submissions Needing Revision' folder to locate your manuscript file.

Please include the following items when submitting your revised manuscript:

- A letter that responds to each point raised by the editor and reviewer(s). You should upload this letter as a separate file labeled 'Response to Reviewers'.
- A marked-up copy of your manuscript that highlights changes made to the original version. You should upload this as a separate file labeled 'Revised Manuscript with Track Changes'.
- An unmarked version of your revised paper without tracked changes. You should upload this as a separate file labeled 'Manuscript'.

Guidelines for resubmitting your figure files are available below the reviewer comments at the end of this letter.

We look forward to receiving your revised manuscript.

Kind regards,

Collins Otieno Asweto, PhD

Academic Editor

PLOS Global Public Health

Journal Requirements:

1. Please provide a detailed online Financial Disclosure statement. This is published with the article. It must therefore be completed in full sentences and contain the exact wording you wish to be published.

a) State the initials, alongside each funding source, of each author to receive each grant. For example: “This work was supported by the National Institutes of Health (####### to AM; ###### to CJ) and the National Science Foundation (###### to AM).”

b) State what role the funders took in the study. If the funders had no role in your study, please state: “The funders had no role in study design, data collection and analysis, decision to publish, or preparation of the manuscript.”

For more information, please go to our submission guidelines:

https://journals.plos.org/globalpublichealth/s/submission-guidelines#loc-financial-disclosure-statement

2. Please ensure that the funders and grant numbers match between the Financial Disclosure field and the Funding Information tab in your submission form. Note that the funders must be provided in the same order in both places as well.

3. Please update your online Competing Interests statement. If you have no competing interests to declare, please state: “The authors have declared that no competing interests exist.”

4. In this instance it seems there may be acceptable restrictions in place that prevent the public sharing of your minimal data. However, in line with our goal of ensuring long-term data availability to all interested researchers, PLOS’ Data Policy states that authors cannot be the sole named individuals responsible for ensuring data access (http://journals.plos.org/globalpublichealth/s/data-availability#loc-acceptable-data-sharing-methods).

Data requests to a non-author institutional point of contact, such as a data access or ethics committee, helps guarantee long term stability and availability of data. Providing interested researchers with a durable point of contact ensures data will be accessible even if an author changes email addresses, institutions, or becomes unavailable to answer requests.

Before we proceed with your manuscript, please also provide non-author contact information (phone/email/hyperlink) for a data access committee, ethics committee, or other institutional body to which data requests may be sent. If no institutional body is available to respond to requests for your minimal data, please consider if there any institutional representatives who did not collaborate in the study, and are not listed as authors on the manuscript, who would be able to hold the data and respond to external requests for data access? If so, please provide their contact information (i.e., email address). Please also provide details on how you will ensure persistent or long-term data storage and availability.

5. Please ensure that you refer to Tables 1, 2 and 3 in your text as, if accepted, production will need these references to link the reader to the tables.

6. If the reviewer comments include a recommendation to cite specific previously published works, please review and evaluate these publications to determine whether they are relevant and should be cited. There is no requirement to cite these works unless the editor has indicated otherwise.

7. Please review your reference list to ensure that it is complete and correct. If you have cited papers that have been retracted, please include the rationale for doing so in the manuscript text, or remove these references and replace them with relevant current references. Any changes to the reference list should be mentioned in the rebuttal letter that accompanies your revised manuscript. If you need to cite a retracted article, indicate the article’s retracted status in the References list and also include a citation and full reference for the retraction notice.

Reviewers' comments:

Reviewer's Responses to Questions

**Comments to the Author**

1. Does this manuscript meet PLOS Global Public Health’s publication criteria?

Reviewer #1: Yes

Reviewer #2: Yes

**********

2. Has the statistical analysis been performed appropriately and rigorously?

Reviewer #1: Yes

Reviewer #2: Yes

**********

3. Have the authors made all data underlying the findings in their manuscript fully available (please refer to the Data Availability Statement at the start of the manuscript PDF file)?

The PLOS Data policy

Reviewer #1: Yes

Reviewer #2: Yes

**********

4. Is the manuscript presented in an intelligible fashion and written in standard English?

Reviewer #1: Yes

Reviewer #2: Yes

**********

Reviewer #1: Kudos to the researchers for this important work. Please consider the following minor concerns:

1. Do you think your current definition of a caregiver as contained in line 110 to 111, and which I assumes is somewhat of your inclusion criteria, is encompassing enough to cover those participants in your study who are not primary caregivers or main decision maker for the children? You might want to modify your definition accordingly.

2. I think reporting your percentages to the nearest one decimal place (e.g. 5.4%) will help ensure consistency in your reporting. For instance, percentage of children who sleep or do not sleep under a mosquito net do not add up to 100, because 'yes' was rounded up to a whole number while 'no' was rounded to one decimal place. I believe the percentage of 'Mother' as main decision maker is incorrect. It is currently listed as 5.8%.

3. In line 184, you mention that majority of caregiver say they could buy the malaria vaccine if it were available for sale. I wonder if you gave them an estimated cost per dose and how many does they will need to get before they responded to this question.

4. Your Table 2 is empty.

Reviewer #2: The manuscript examines caregivers’ willingness to accept the malaria vaccine for under-five children in Uganda prior to national rollout, while also identifying factors associated with vaccine acceptance. The study addresses an important implementation-relevant question in a high-burden setting (Sub-Saharan Africa) and is well-positioned within the existing literature on vaccine acceptance and hesitancy, addressing a clear evidence gap regarding malaria vaccine readiness immediately before implementation.

The authors employ a cross-sectional design, enrolling caregivers at a referral hospital and using interviewer-administered questionnaires. The use of the Kish-Leslie formula for sample size estimation and modified Poisson regression to estimate prevalence ratios is appropriate for the stated research question and outcome prevalence. Ethical approval and informed consent procedures are adequately described. Overall, the methods are technically sound and sufficiently detailed to allow reproducibility. The results are clearly presented and generally support the authors’ conclusions. The finding of high willingness to vaccinate despite low baseline awareness is notable and policy relevant. Associations between willingness and education level, prior AEFI experience, and perceptions of vaccine safety and effectiveness are supported by the data presented in the tables.

This study is a well-conducted, timely, and policy-relevant research conducted before vaccine rollout, using appropriate statistical methods for the study design and outcome, and having a clear linkage between findings and implications for malaria vaccine implementation, but there remains a risk of response bias during data collection and concerns with generalizability due to a largely urban and hospital-based sample. With minor clarifications and strengthened discussion around key findings and limitations, the manuscript would be suitable for publication.

Major Issues

1. The manuscript should more explicitly discuss potential biases introduced by providing information (“orientation”) about the vaccine during data collection, which may have influenced willingness responses and introduced potential response bias.

2. Variable denominators across some analyses suggest missing data or eligibility differences. The denominator (n) for each group appears to change from variable to variable, for example, in Table 3, IPTp analyses are based on 37 participants rather than 43 in the “not willing to vaccinate” group, and on 213 rather than 247 in the “willing to vaccinate” group. Clarification or justification should be provided.

3. Some findings, particularly the lower willingness among caregivers with tertiary education, seem counterintuitive and would benefit from deeper interpretation or hypothesized mechanisms grounded in existing literature. Only one reference was provided to support the strength of this claim. Perhaps digging more into existing literature would provide a better standing for this finding.

Minor issues

1. As data were collected in a referral hospital setting, concerns with generalizability to rural or community-based populations should be more clearly acknowledged.

2. Some clarification is needed regarding the measurement of adverse events following immunization (AEFI), specifically whether these were caregiver-reported or clinically verified, and whether severity was assessed

3. References are duplicated from line 409

**********

what does this mean?). If published, this will include your full peer review and any attached files.

**Do you want your identity to be public for this peer review?**  If you choose “no”, your identity will remain anonymous but your review may still be made public.

For information about this choice, including consent withdrawal, please see our Privacy Policy

Reviewer #1: No

Reviewer #2: **Yes:**  Oyinkansola Taiwo

**********

[NOTE: If reviewer comments were submitted as an attachment file, they will be attached to this email and accessible via the submission site. Please log into your account, locate the manuscript record, and check for the action link "View Attachments". If this link does not appear, there are no attachment files.]

# Author comment: Author response to Decision Letter 1

**DOI:** 10.1371/journal.pgph.0005764.r002

9 Feb 2026

Attachment Submitted filename: Response to reviewers.docx

# Peer review: Decision Letter 1

**DOI:** 10.1371/journal.pgph.0005764.r003

**Authors:** Jennifer Tucker

20 Apr 2026

PGPH-D-25-03668R1

Caregivers’ Willingness and Factors Influencing Uptake of the Malaria Vaccine Among Under-Five Children at Masaka Regional Referral Hospital, Uganda, March 2025

PLOS Global Public Health

Dear Dr. Namusisi,

Thank you for submitting your manuscript to PLOS Global Public Health. After careful consideration, we feel that it has merit but does not fully meet PLOS Global Public Health’s publication criteria as it currently stands. Therefore, we invite you to submit a revised version of the manuscript that addresses the points raised during the review process.

The manuscript has been evaluated by two reviewers, and their comments are available below.

The reviewers have raised a number of major concerns. They feel the manuscript requires updating due to some misreported data in text, presentation of the conceptual model could be strengthened and a number of other revisions have been recommended.

Could you please carefully revise the manuscript to address all comments raised?

Please submit your revised manuscript by Jun 04 2026 11:59PM. If you will need more time than this to complete your revisions, please reply to this message or contact the journal office at globalpubhealth@plos.org. When you're ready to submit your revision, log on to https://www.editorialmanager.com/pgph/ and select the 'Submissions Needing Revision' folder to locate your manuscript file.

Please include the following items when submitting your revised manuscript:

- A letter that responds to each point raised by the editor and reviewer(s). You should upload this letter as a separate file labeled 'Response to Reviewers'.
- A marked-up copy of your manuscript that highlights changes made to the original version. You should upload this as a separate file labeled 'Revised Manuscript with Track Changes'.
- An unmarked version of your revised paper without tracked changes. You should upload this as a separate file labeled 'Manuscript'.

Guidelines for resubmitting your figure files are available below the reviewer comments at the end of this letter.

As the corresponding author, your ORCID iD is verified in the submission system and will appear in the published article. PLOS supports the use of ORCID, and we encourage all coauthors to register for an ORCID iD and use it as well. Please encourage your coauthors to verify their ORCID iD within the submission system before final acceptance, as unverified ORCID iDs will not appear in the published article. *Only*  the individual author can complete the verification step; PLOS staff *cannot*  verify ORCID iDs on behalf of authors.

We look forward to receiving your revised manuscript.

Kind regards,

Jennifer Tucker, PhD

Staff Editor

PLOS Global Public Health

Journal Requirements:

If the reviewer comments include a recommendation to cite specific previously published works, please review and evaluate these publications to determine whether they are relevant and should be cited. There is no requirement to cite these works unless the editor has indicated otherwise.

Additional Editor Comments (if provided):

[Note: HTML markup is below. Please do not edit.]

Reviewers' comments:

Reviewer's Responses to Questions

**Comments to the Author**

Reviewer #3: (No Response)

Reviewer #4: (No Response)

**********

publication criteria?

Reviewer #3: Yes

Reviewer #4: Yes

**********

3. Has the statistical analysis been performed appropriately and rigorously?

Reviewer #3: Yes

Reviewer #4: No

**********

4. Have the authors made all data underlying the findings in their manuscript fully available (please refer to the Data Availability Statement at the start of the manuscript PDF file)?

The PLOS Data policy

Reviewer #3: No

Reviewer #4: No

**********

5. Is the manuscript presented in an intelligible fashion and written in standard English?

Reviewer #3: Yes

Reviewer #4: Yes

**********

Reviewer #3: Reviewer Recommendations and Comments

Reviewer:

LAST NAME: DEMES

FIRST NAME: JOSEPH ADRIEN EMMANUEL

Title of article:

Caregivers’ Willingness and Factors Influencing Uptake of the Malaria Vaccine Among Under-Five Children at Masaka Regional Referral Hospital, Uganda, March 2025

Major comments:

The authors should be commended for focusing on an understudied topic: Caregivers’ Willingness and Factors Influencing Uptake of the Malaria Vaccine Among Under-Five Children.

It would strengthen the manuscript to present a theoretical or conceptual model in the introduction or literature review. A diagram illustrating the relationships between key variables could help guide the interpretation of the findings, clarify causal pathways, and support the identification of potential confounding factors. Such a framework would also assist in justifying variable selection and analytical choices.

Similarly, the inclusion and exclusion criteria coould be described in greater detail to enhance transparency and reproducibility, and to allow readers to better assess potential selection bias.

The issue of missing data requires further clarification. The authors could indicate:

• Whether missing data were present, and for which variables

• The extent and patterns

• The methods used to handle missing data

• The potential influence of missing data on the study outcomes and conclusions.

From an analytical perspective, the authors could report whether there is an estimate of variability within each group, and whether variance is comparable across groups.

In addition, the role of social determinants of health warrants deeper exploration. Specifically:

• Are there observable differences between socio-economic groups (e.g., poorest vs. wealthiest quintiles, geographic disparities) (is the willingness and awareness different, taking into account the geographic disparities?

• How were social inequalities and disparities incorporated into the analysis?

• What implications do these disparities have for the interpretation of the findings?

Finally, the authors could more explicitly address potential sources of bias that could influence the direction or magnitude of the results. This includes, but is not limited to:

• Selection bias;

• Design-related bias;

• Measurement or instrumentation bias;

• Analytical bias.

The manuscript could describe the strategies used to minimize or control bias, including how major confounding variables were identified and managed.

It could be interesting to find a way to make it easy for the readers to understand the paper coherently.

What do you think?

Regarding the methodology, what justifies the Poisson regression and the systematic sampling? Did you have a sampling frame or a list?

Also, review the whole document structure and flow, and coherence so that everything could have a thread from the beginning to the end.

Minor comments:

The language of the document requires a comprehensive review. Professional proofreading and editing of the entire document are strongly recommended. Furthermore, a significant structural revision is necessary to enhance overall flow and coherence.

Were previous studies similar to this study, published in the scientific literature?

• What were the major findings?

• How is your study similar to or different from those studies?

What are the policy implications of your study? Could you integrate this aspect into the discussion section?

Decision: A minor revision is needed before publication.

Reviewer #4: Recommendation

Major Revision Required.

The study addresses a genuinely important and timely public health question, and several of the previous reviewers' concerns have been addressed. However, the factual error in the abstract, the validity concerns surrounding the post-orientation willingness measure, the incomplete reporting of the analytical model, and the unresolved ethics approval question must be adequately addressed before this manuscript can be considered for publication. I encourage the authors to engage thoroughly with each point raised and to revise accordingly.

**********

what does this mean?). If published, this will include your full peer review and any attached files.

**Do you want your identity to be public for this peer review?**  If you choose “no”, your identity will remain anonymous but your review may still be made public.

For information about this choice, including consent withdrawal, please see our Privacy Policy

Reviewer #3: **Yes:**  Joseph Adrien Emmanuel Demes

Reviewer #4: No

**********

[NOTE: If reviewer comments were submitted as an attachment file, they will be attached to this email and accessible via the submission site. Please log into your account, locate the manuscript record, and check for the action link "View Attachments". If this link does not appear, there are no attachment files.]

Attachment Submitted filename: Comments of Reviewer.docx

Attachment Submitted filename: PEER REVIEW_HD.docx

# Author comment: Author response to Decision Letter 2

**DOI:** 10.1371/journal.pgph.0005764.r004

22 Jun 2026

Attachment Submitted filename: Response to reviewers June-N.pdf

# Peer review: Decision Letter 2

**DOI:** 10.1371/journal.pgph.0005764.r005

**Authors:** Mila Nu Nu Htay

20 Jul 2026

PGPH-D-25-03668R2

Caregivers’ Willingness and Factors Influencing Uptake of the Malaria Vaccine Among Under-Five Children at Masaka Regional Referral Hospital, Uganda

PLOS Global Public Health

Dear Dr. Namusisi,

Thank you for submitting your manuscript to PLOS Global Public Health. After careful consideration, we feel that it has merit but does not fully meet PLOS Global Public Health’s publication criteria as it currently stands. Therefore, we invite you to submit a revised version of the manuscript that addresses the points raised during the review process.

Please submit your revised manuscript by Aug 19 2026 11:59PM. If you will need more time than this to complete your revisions, please reply to this message or contact the journal office at globalpubhealth@plos.org. When you're ready to submit your revision, log on to https://www.editorialmanager.com/pgph/ and select the 'Submissions Needing Revision' folder to locate your manuscript file.

Please include the following items when submitting your revised manuscript:

- A letter that responds to each point raised by the editor and reviewer(s). You should upload this letter as a separate file labeled 'Response to Reviewers'.
- A marked-up copy of your manuscript that highlights changes made to the original version. You should upload this as a separate file labeled 'Revised Manuscript with Track Changes'.
- An unmarked version of your revised paper without tracked changes. You should upload this as a separate file labeled 'Manuscript'.

Guidelines for resubmitting your figure files are available below the reviewer comments at the end of this letter.

As the corresponding author, your ORCID iD is verified in the submission system and will appear in the published article. PLOS supports the use of ORCID, and we encourage all coauthors to register for an ORCID iD and use it as well. Please encourage your coauthors to verify their ORCID iD within the submission system before final acceptance, as unverified ORCID iDs will not appear in the published article. *Only*  the individual author can complete the verification step; PLOS staff *cannot*  verify ORCID iDs on behalf of authors.

We look forward to receiving your revised manuscript.

Kind regards,

Mila Nu Nu Htay, phd

Academic Editor

PLOS Global Public Health

Journal Requirements:

If the reviewer comments include a recommendation to cite specific previously published works, please review and evaluate these publications to determine whether they are relevant and should be cited. There is no requirement to cite these works unless the editor has indicated otherwise.

Please review your reference list to ensure that it is complete and correct. If you have cited papers that have been retracted, please include the rationale for doing so in the manuscript text, or remove these references and replace them with relevant current references. Any changes to the reference list should be mentioned in the rebuttal letter that accompanies your revised manuscript. If you need to cite a retracted article, indicate the article’s retracted status in the References list and also include a citation and full reference for the retraction notice.

Additional Editor Comments (if provided):

[Note: HTML markup is below. Please do not edit.]

Reviewers' comments:

Reviewer's Responses to Questions

**Comments to the Author**

Reviewer #3: All comments have been addressed

**********

publication criteria?

Reviewer #3: Yes

**********

3. Has the statistical analysis been performed appropriately and rigorously?

Reviewer #3: Yes

**********

4. Have the authors made all data underlying the findings in their manuscript fully available (please refer to the Data Availability Statement at the start of the manuscript PDF file)?

The PLOS Data policy

Reviewer #3: Yes

**********

5. Is the manuscript presented in an intelligible fashion and written in standard English?

Reviewer #3: Yes

**********

Reviewer #3: No comments

**********

what does this mean?). If published, this will include your full peer review and any attached files.

**Do you want your identity to be public for this peer review?**  If you choose “no”, your identity will remain anonymous but your review may still be made public.

For information about this choice, including consent withdrawal, please see our Privacy Policy

Reviewer #3: **Yes:**  Joseph Adrien Emmanuel Demes

**********

[NOTE: If reviewer comments were submitted as an attachment file, they will be attached to this email and accessible via the submission site. Please log into your account, locate the manuscript record, and check for the action link "View Attachments". If this link does not appear, there are no attachment files.]

Attachment Submitted filename: 24 June 26_Comments of Reviewer.docx

# Author comment: Author response to Decision Letter 3

**DOI:** 10.1371/journal.pgph.0005764.r006

21 Aug 2026

Attachment Submitted filename: Response to reviewers.pdf

# Editor report: Decision Letter 3

**DOI:** 10.1371/journal.pgph.0005764.r007

**Authors:** Mila Nu Nu Htay

14 Sep 2026

Caregivers’ Willingness and Factors Influencing Uptake of the Malaria Vaccine Among Under-Five Children at Masaka Regional Referral Hospital, Uganda

PGPH-D-25-03668R3

Dear Ms Namusisi,

We are pleased to inform you that your manuscript 'Caregivers’ Willingness and Factors Influencing Uptake of the Malaria Vaccine Among Under-Five Children at Masaka Regional Referral Hospital, Uganda' has been provisionally accepted for publication in PLOS Global Public Health.

Before your manuscript can be formally accepted you will need to complete some formatting changes, which you will receive in a follow up email. A member of our team will be in touch with a set of requests.

Please note that your manuscript will not be scheduled for publication until you have made the required changes, so a swift response is appreciated.

IMPORTANT: The editorial review process is now complete. PLOS will only permit corrections to spelling, formatting or significant scientific errors from this point onwards. Requests for major changes, or any which affect the scientific understanding of your work, will cause delays to the publication date of your manuscript.

If your institution or institutions have a press office, please notify them about your upcoming paper to help maximize its impact. If they'll be preparing press materials, please inform our press team as soon as possible -- no later than 48 hours after receiving the formal acceptance. Your manuscript will remain under strict press embargo until 2 pm Eastern Time on the date of publication. For more information, please contact globalpubhealth@plos.org.

Thank you again for supporting Open Access publishing; we are looking forward to publishing your work in PLOS Global Public Health.

Best regards,

Mila Nu Nu Htay, phd

Academic Editor

PLOS Global Public Health

***********************************************************

Reviewer Comments (if any, and for reference):
